# Developmental pattern and structural factors of dendritic survival in cerebellar granule cells *in vivo*

**DOI:** 10.1038/s41598-018-35829-y

**Published:** 2018-12-03

**Authors:** Matasha Dhar, Adam W. Hantman, Hiroshi Nishiyama

**Affiliations:** 1 Center for Learning and Memory, Department of Neuroscience, The University of Texas at Austin, 1 University Station Stop, C7000 Austin, Texas USA; 2 Janelia Research Campus, Howard Hughes Medical Institute, Ashburn, Virginia USA

## Abstract

Granule cells (GCs) in the cerebellar cortex are important for sparse encoding of afferent sensorimotor information. Modeling studies show that GCs can perform their function most effectively when they have four dendrites. Indeed, mature GCs have four short dendrites on average, each terminating in a claw-like ending that receives both excitatory and inhibitory inputs. Immature GCs, however, have significantly more dendrites—all without claws. How these redundant dendrites are refined during development is largely unclear. Here, we used *in vivo* time-lapse imaging and immunohistochemistry to study developmental refinement of GC dendritic arbors and its relation to synapse formation. We found that while the formation of dendritic claws stabilized the dendrites, the selection of surviving dendrites was made before claw formation, and longer immature dendrites had a significantly higher chance of survival than shorter dendrites. Using immunohistochemistry, we show that glutamatergic and GABAergic synapses are transiently formed on immature GC dendrites, and the number of GABAergic, but not glutamatergic, synapses correlates with the length of immature dendrites. Together, these results suggest a potential role of transient GABAergic synapses on dendritic selection and show that preselected dendrites are stabilized by the formation of dendritic claws—the site of mature synapses.

## Introduction

The specific pattern of a neuronal dendritic arbor determines how that neuron integrates synaptic inputs from multiple, diverse presynaptic cells^1–3^. Defects in dendritic refinement result in inappropriate synaptic connectivity and integration leading to impaired brain function seen in various neurological disorders such as: schizophrenia, Down’s syndrome, fragile X syndrome, Angelman’s syndrome, Rett’s syndrome, and autism spectrum disorder^4,5^.

Decades of research has shown that dendritic arborization starts with an exuberant phase of growth, followed by the pruning of extraneous dendrites, and subsequent maturation of surviving dendrites^6,7^. Many cell-intrinsic and extrinsic factors have been identified as regulators of this process^6,8^. However, because conventional experimental approaches relied on snapshot images collected from fixed tissue samples, it remains unclear, especially in the mammalian brain, how individual dendritic arbors are selectively stabilized or pruned *in vivo*.

The cerebellar granule cells (GCs) are ideal model neurons for studying the spatiotemporal pattern of dendritic refinement *in vivo* and revealing the factors that determine stabilization of individual dendrites. This is because, in mice, the majority of GCs undergo dendritic development during the second postnatal week^9^, allowing for *in vivo* investigation of this phenomenon using time-lapse imaging. Mature GCs have a simple dendritic arbor consisting of 3–5 short dendrites, each of which has a distinctly identifiable postsynaptic claw-like structure at the tip^10^. They receive an excitatory and an inhibitory input on each claw from a mossy fiber (MF) and a Golgi cell axon, respectively. The simplicity and compactness of this arbor combined with the ease of identification of the synapses are highly favorable for following the development of individual dendrites and its relationship with the formation of the claw (the site for synaptic contact on these neurons). Recent studies show that the size and pattern of the GC dendritic arbor are key for its function in the cerebellar circuitry^11^. Computational analysis shows that GCs allow high-dimensional representation of incoming sensorimotor information, conveyed by MFs, most efficiently when each GC has four dendrites^12^. Importantly, MFs do not undergo structural plasticity in adult rats, suggesting that MF-GC synapses are hard-wired^13^. Therefore, the establishment of appropriate GC arbors during development is vital for its functioning throughout an animal’s lifespan.

To study GC dendritic refinement, we used the TCGO transgenic mice in which GCs are sparsely labeled with mCitrine, a variant of yellow fluorescent protein^14,15^. Using daily *in vivo* imaging, we analyzed developmental remodeling of GC dendritic arbors and found that dendritic refinement completed with the formation of a claw-like ending. Once a claw was formed on a dendrite, its motility was significantly reduced and was rarely pruned afterward. However, the final surviving dendrites were selected prior to the formation of the claw, and longer immature dendrites had a higher chance of survival. Since synapse formation has been shown to be important for dendritic arborization, we used immunohistochemistry to analyze putative glutamatergic and GABAergic synaptic sites on immature dendrites. We found that the number of transient GABAergic synapses, but not glutamatergic synapses, was positively correlated with the length of immature dendrites. These results suggest that transient GABAergic inputs to immature dendrites may play a role in their survival, and that the formation of a claw-like ending permanently stabilizes the dendrites.

## Results

### Time-lapse imaging of dendritic refinement in cerebellar granule cells (GCs)

GCs proliferate postnatally in the external GC layer, the most superficial layer of the developing cerebellar cortex^16^. As proliferating GCs exit the cell cycle, they start migrating down radially toward the internal GC layer, and the external GC layer eventually disappears. In rats, the onset of radial migration peaks around postnatal day (P) 8–11, and the majority of GCs reach the internal GC layer around P10–14 where they initiate dendritic development^9^. Previous studies have shown that nascent dendritic arbors contain excess dendrites that are subsequently pruned, but how surviving dendrites are selected and subsequently stabilized in the intact synaptic circuit is poorly understood^17–19^. To study this dendritic refinement process *in vivo*, we implanted cranial windows over cerebellar lobules 6–7 of TCGO transgenic mice and performed *in vivo* time-lapse imaging from P11 to at least P23. Although some GCs receive excitatory inputs from unipolar brush cells, the density of unipolar brush cells is low in lobules 6–7; thus, GCs imaged in this study receive excitatory inputs predominantly from MFs. A total of 21 developing GCs were imaged from 6 pups, out of which 7 cells were imaged from P11 onwards, 9 from P12 onwards, 2 from P13 onwards and 3 from P14 onwards. GCs were selected as they appeared in the internal granule cell layer under the cranial window. All imaged GCs had supernumerary dendrites without claw-like endings at the first imaging time point.

**Figure 1:**
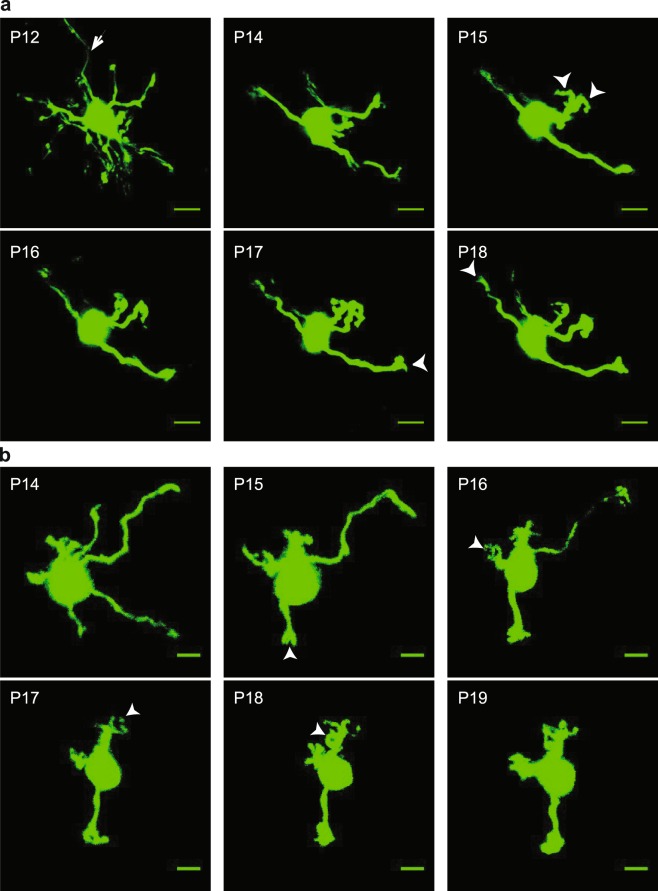
Time-lapse imaging of developing cerebellar granule cells *in vivo*. (**a**,**b**) Each image is a maximum projection of a 3-D stack of labeled GCs imaged *in vivo*. Each series (**a**,**b**) shows a GC developing with its developmental age noted in the upper-left of the image (P, postnatal day). Arrows indicate a parallel fiber axon emerging from the soma and arrowheads indicate the appearance of a claw on a dendrite. Scale bar is 5 µm.

Repeated *in vivo* imaging allowed us to quantify how individual GCs sequentially develop their dendritic arbors for the first time in the intact synaptic circuits (Fig. 1a,b). First, we measured the total number of dendrites, total length of dendrites (including claw length as it appeared on a dendrite), and appearance of a claw-like structure on any dendrite for each imaging time point. We then identified the pruning, growth and claw formation phases of GC dendritic development with respect to the age of the animals (Supplementary Fig. S1). We found that developing GCs underwent (1) a loss in the number of dendrites (i.e., pruning phase from P11–17), (2) an increase in total dendritic length (i.e., maturation phase from P14–19), and (3) claw formation until P21. These data represent the overall developmental profile of a GC population and suggest that different phases of dendritic refinement—pruning, maturation, and synapse formation—occur in succession with slightly different time windows.

### Dendritic pruning and maturation occur simultaneously within individual GCs

**Figure 2:**
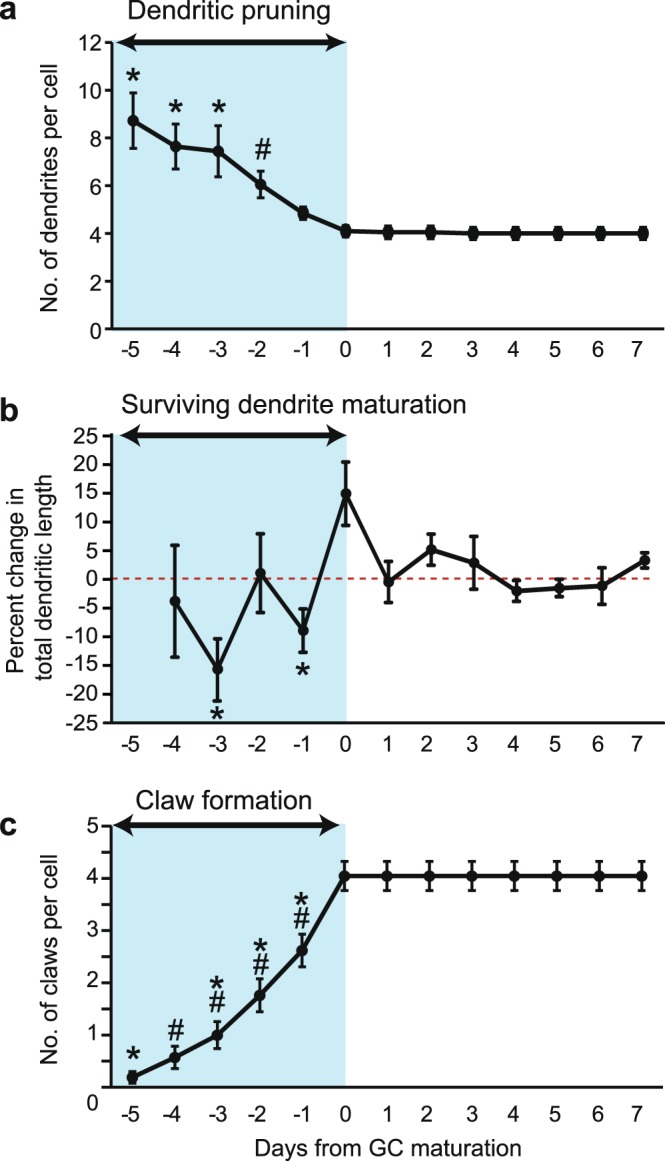
Pattern of GC dendritic development *in vivo* with respect to cell age. (**a**) Average number of total dendrites per cell ( ± SEM) at each time point (n = 21 cells from 6 mice). Time point 0 is the day when a GC attained mature morphology. The blue box highlights the dendritic pruning phase for each GC imaged. Number of dendrites per cell were significantly different over time (One-way ANOVA with Tukey post-hoc analysis: F (12, 229) = 10.12, p < 0.0001, *p < 0.001 for time points −5, −4, −3 compared with time points −1 to 7, #p < 0.05 for time point −2 compared with time points 3–7). (**b**) Average percent change in the total dendritic length per cell (±SEM) at each time point. The blue box highlights the dendritic maturation phase for each GC imaged. The percent change in total dendritic length was significantly different over time (One-way ANOVA with Tukey analysis: F (9, 150) = 2.942, p = 0.003, *p < 0.01 for time points −3, −1 compared with time points 0, 2. (**c**) Average number of claws per cell (±SEM) at each time point. The blue box highlights the claw formation phase for each GC imaged. Number of claws per cell was significantly different over time (One-way ANOVA with Tukey analysis: F (12, 260) = 32.21, p < 0.0001, *p < 0.001 for time point −5 compared with time points −2 to 7, #p < 0.01 for time point −4 compared with time points −1 to 7, *#p < 0.01 for time point −3 compared with time points −1 to 7, *#p < 0.05 for time points −2, −1 compared to time points 0 to 7).

The developmental profile of neuronal populations is traditionally studied using snapshot images of fixed tissue specimens taken from different animals. Such studies support the model that different phases of dendritic refinement occur in succession. However, since the timing of birth and dendritic development differ among GCs, aligning the time-lapse images by the age of the animals may obscure the developmental pattern of individual GCs. We therefore realigned the same data shown in Supplementary Fig. S1 by the developmental status of each GC. We defined “day 0” as the day when a GC forms claws on all of its dendrites (Fig. 2). Approximately one dendrite was pruned each day in individual GCs from the day −5 to day 0 (Fig. 2a). No pruning occurred after day 0, indicating that the dendritic arbor of GCs reaches the mature state when claws are formed on all of the dendrites (Fig. 2a). As is evident from our time-lapse imaging data, pruning of some dendrites proceeds simultaneously with maturation of others (Fig. 1). In order to determine the timing of dendritic maturation for individual GCs, we quantified the daily change in the total dendritic length, because dendritic maturation is associated with retraction and elongation of surviving dendrites. Since the total dendritic length varies among different GCs, simply averaging the length across the GCs may not precisely represent how individual GCs develop. To consider the cell-to-cell variation, we quantified the daily rate of change—percent change in the total dendritic length compared to the previous day—in individual GCs (Fig. 2b). The average daily rate of change showed that significant changes in individual GCs occurred only before day 0, suggesting that dendritic maturation phase is over at the end of the pruning phase. Claw formation occurred throughout the pruning phase with the majority of claws (3/4 final claws) on a cell being added within 2 days from maturation (Fig. 2c). These data indicate that the different aspects of dendritic refinement (i.e., pruning of excess dendrites, maturation of surviving dendrites, and formation of dendritic claws), proceed simultaneously in individual GCs, and that the refinement process completes when claws are formed on all of the dendrites.

### Role of claw formation in dendritic selection and stabilization

Dendritic claws are the site of synaptic contacts in both mature and developing GCs^20,21^. Since synaptic contacts and activity have been shown to be important for dendritic growth and maturation^7^, we examined the role of claw formation in dendritic refinement. To do so, we first examined how much the final pattern of dendritic arbors is determined before claws are formed. Conventionally, mature GCs are described to have 3–5 dendrites emerging from the soma. However, a recent study showed that nearly half of mature GCs had branched dendrites; a set of two dendrites that consists of one primary dendrite (emerging from the soma) and one secondary dendrite (emerging from the primary dendrite)^11^. The same study also showed that GCs with branched dendrites (branched GCs) receive more MF inputs than GCs with unbranched dendrites (unbranched GCs), suggesting that the branched GCs and unbranched GCs have a different degree of significance in neuronal computation in the GC layer.

**Figure 3:**
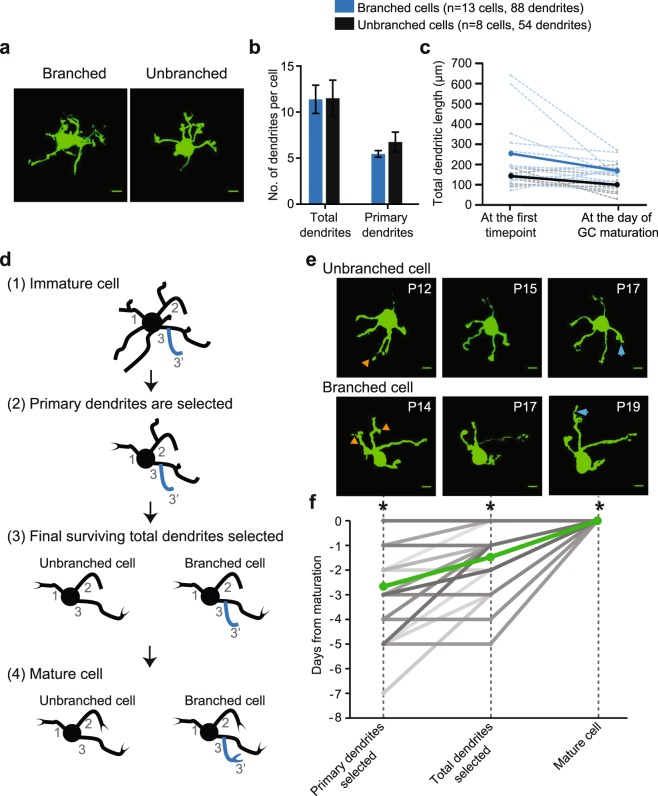
Effect of claw formation on dendritic patterning and selection in branched and unbranched GCs *in vivo*. (**a**) Maximum projection images of immature P12 GCs with branched dendrites at this stage. Upon maturation, the left GC became a branched GC, and the right GC became an unbranched GC. (**b**) Average number of total and primary dendrites (±SEM) for branched and unbranched GCs at their first time point (immature). (**c**) Average total dendritic length (±SEM) for branched and unbranched GCs at their first time point and the day of their maturation shown in solid blue and black lines, respectively. Dashed blue and black lines are individual GCs. Total dendritic length is significantly different at the first time point (T-test, p = 0.02) and the day of maturation (T-test, p = 0.01) between branched and unbranched GCs. Total dendritic length is slightly reduced from first time point to the day of maturation for both branched and unbranched GCs (Branched GC: Paired t-test, p = 0.042; Unbranched GC: Paired t-test, p = 0.048). (**d**) Schematic describing the stages of GC dendritic selection for branched and unbranched cells starting with an (1) immature cell with supernumerary primary and secondary dendrites, followed by the three time points identified in the text. The three surviving primary dendrites are labeled 1, 2 and 3. In the case of a branched cell, at least one secondary dendrite (3′) will also survive. (**e**) Projection images of two GCs: unbranched (top) and branched (bottom) displaying the three stages of dendritic selection. Orange arrowheads mark the extraneous secondary dendrites pruned, and blue arrows mark the appearance of the claw. (**f**) Days from GC maturation when a GC attains the defined stage in the dendritic selection process, i.e., primary dendrite selection, total dendrite selection, mature cell. Each gray line is an individual GC, and the green line is the average (n = 21 GCs). The day of primary dendrite selection is significantly different from the other time points (Repeated measure ANOVA with Tukey analysis: F(2,20,40) = 25.85, p < 0.0001, *p < 0.001 amongst all groups). Scale bar is 5 µm.

Amongst 21 GCs we imaged *in vivo*, there were 13 branched GCs and 8 unbranched GCs at P23. Similar to the previous report, we found that the total number of dendrites was higher in branched GCs than unbranched GCs, while the number of primary dendrites was the same amongst these groups (Supplementary Fig. S2a,b). However, at the earliest immature stage, all of them had a similar number of primary and total dendrites regardless of whether the GCs eventually became unbranched or branched (Fig. 3a,b). To determine when branched and unbranched GCs start to differentiate from each other, we quantified the change in the total dendritic length because the total dendritic length is significantly different between branched and unbranched GCs after maturation (Supplementary Fig. S2c). Branched GCs had significantly longer total dendritic length when compared to unbranched GCs at all critical timepoints: at the first imaging time point, at the day of GC maturation and at the last time point imaged (Fig. 3c and Supplementary Fig. S2c). These data suggest that the eventual fate of immature GCs, whether they become unbranched or branched, is determined to some extent early in the dendritic refinement process; immature GCs with longer dendrites tend to become branched GCs. This result suggests that the dendritic selection process begins in immature dendrites before claws are formed.

To further examine the relationship between dendritic selection and claw formation, we determined the temporal pattern of dendritic branch selection in relation to claw formation. For each GC we identified three time points that an immature GC go through during maturation (Fig. 3d). The first time point is the day when final surviving primary dendrites are selected, i.e., all excess primary dendrites have been pruned such that the remaining primary dendrites are the only ones surviving in the mature cells. Excess secondary dendrites may still exist at this time point. The second time point is the day when all final surviving dendrites are selected, i.e., all supernumerary dendrites are pruned. In the case of an unbranched GC, only the selected primary dendrites will remain, while in the case of a branched GC, the selected primary and secondary dendrites will remain at this time point. The third time point is the day when claws are present on all the surviving dendrites of the GC. We defined the third time point as day ‘0’ and quantified the temporal relationship between these time points. We found that the selection of surviving primary dendrites was completed on an average of 2.67 ± 0.46 days before claws appeared on all the dendrites (Fig. 3e,f). As shown in Fig. 2c, only about one claw is formed per GC at this time point, indicating that the final surviving primary dendrites are determined significantly before MF-GC synapses mature. The selection of all surviving dendrites was completed on average 1.24 ± 0.34 days after the selection of primary dendrites, and an average of 1.43 ± 0.32 days before claws appeared on all the dendrites (Fig. 3f). There was no significant difference in these time points between branched and unbranched GCs (Primary dendrite selection timepoint ± SEM: branched = −2.76 ± 0.54, unbranched = −2.5 ± 0.86, p = 0.39; Total dendrite selection timepoint ± SEM: branched = −1.76 ± 0.44, unbranched = −0.88 ± 0.39, p = 0.09). Importantly, nearly half of the dendrites do not yet form claws at this time point (Fig. 2c). These results indicate that, in most GCs, the selection of primary as well as secondary dendrites (in case of branched GCs) precedes claw formation and suggest that synapse formation at claws has a minor, if any, contribution to the selection of surviving dendrites.

**Figure 4:**
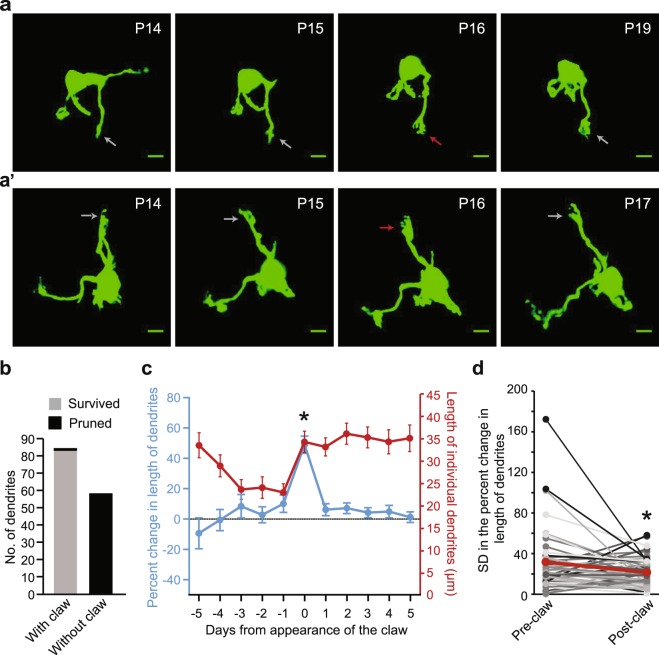
Effect of claw formation on the survival and growth of individual GC dendrites. (**a**,a’) Maximum projection images of 3-D stacks of two labeled GCs. Red arrows mark the appearance of the claw on the dendrite, and gray arrows mark the same dendrite in images from days preceding or following this event. (**b**) Number of primary dendrites with a claw (n = 84) or without claws (n = 58) that survived or were pruned. (**c**) Average percent change in the length of individual dendrites (±SEM, blue) and average length of individual dendrites (±SEM, red) at each time point, with time point 0 being the day a claw was observed on a dendrite. The percent change in length of individual dendrites was significantly different over time (Kruskal-Wallis non-parametric ANOVA with Dunn’s multiple comparison, H (11) = 62.59, p = 0.0001, *p < 0.0001 for time point 0 compared to all other time points). The length of individual dendrites was significantly different over time (Kruskal-Wallis non-parametric ANOVA with Dunn’s multiple comparison, H (11) = 67.74, p = 0.0001, *p < 0.01 for time point −3, −2 and −1 compared to time points 0–5). (**d**) Standard deviation (SD) in the percent change values of individual dendrites prior to (before day 0 from panel c) and after (following day 0 from panel c) claw formation. Each individual dendrite measured is shown in shades of gray, and the average is shown in red. The standard deviation of percent changes in individual dendrites is significantly reduced after claw formation (Paired T-test, *p = 0.015).

Even though claw formation may not contribute to dendritic selection, it may still be important for final stabilization of the selected dendrites. To understand the effect of claw formation on dendritic stabilization, we examined the temporal relationship between claw formation and dendritic survival and length (Fig. 4). Out of 142 total primary dendrites imaged at the first day of time-lapse imaging, 84 dendrites developed a claw subsequently, while 58 dendrites never matured to that stage. Out of 84 dendrites that developed a claw, only one was eliminated, while all of the 58 dendrites that never developed a claw were eliminated (Fig. 4b). Thus, whether a dendrite develops a claw or not is almost perfectly related to whether it survives or disappears. Individual dendrites, irrespective of if they were pruned or survived, show day-to-day fluctuation in their length (Supplementary Fig. S3). Nearly 90% of all surviving dendrites showed an increase in their length on the day of claw formation, with 60% showing their largest growth throughout the imaging series (Fig. 4c, Supplementary Fig. S3c,d).

These results suggest that claw formation is a major, energy-intensive event in the lifetime of a GC dendrite. Furthermore, the sudden growth of individual dendrites at the day of claw formation indicates that each claw is formed rapidly once the formation starts (Fig. 4c, Supplementary Fig. S3c,d). Moreover, the day-to-day fluctuation in dendritic length was significantly reduced after claw formation, resulting in more stable mature dendrites (Fig. 4d). Together, these data suggest that claw formation is a landmark event in GC dendritic refinement in that it stabilizes the dendrites that are already selected to survive and finalizes the arborization of GC dendrites.

### Transient appearance of glutamatergic and GABAergic synapses on dendritic shafts

**Figure 5:**
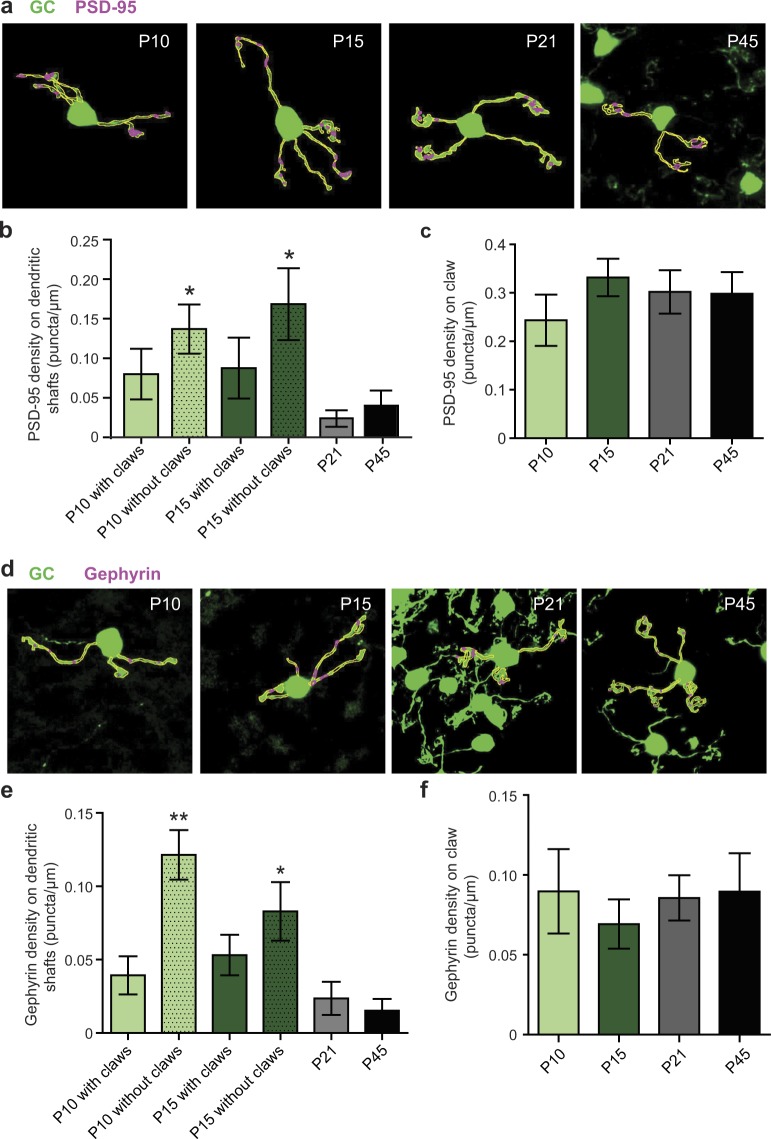
Immunohistochemical analysis of the developmental pattern of excitatory and inhibitory synapse formation on GC dendrites. (**a**) Representative images of GCs from animals of different ages showing the method used for PSD-95 puncta analysis. A ROI (shown in yellow outline) was traced around the dendrite and claw (separately, if present) of the imaged GC and number of PSD-95 puncta (puncta > 0.5 µm, shown in magenta outline) were measured within the ROI. The analysis was performed on z-stacks; the represented image is the maximum projection of the analyzed images. Scale bar is 5 µm. (**b**) Average PSD-95 density on dendritic shafts (±SEM) in animals of different ages (n = number of dendritic shafts collected from at least 3 animals per age group). PSD-95 density is significantly different amongst dendritic shafts of different age groups (Kruskal-Wallis non-parametric ANOVA, H (6) = 21.53, p = 0.0006, *p < 0.05 for P10 and P15 dendrites without claws compared to P21 and P45 dendritic shafts, all with claws). (**c**) Average PSD-95 density on claws (±SEM) in animals of different ages (n = number of claws collected from at least 3 animals per age group). (**d**) Representative images of GCs from animals of different ages showing the method used for Gephyrin puncta analysis, which was the same as the PSD-95 analysis described in a. (**e**) Average Gephyrin density on dendritic shafts (±SEM) in animals of different ages (n = number of dendritic shafts collected from at least 3 animals per age group). Gephyrin density is significantly different amongst dendritic shafts of different age groups (Kruskal-Wallis non-parametric ANOVA, H (6) = 37.36, p < 0.0001, **p < 0.05 for P10 without claws compared to P10 with claws, P21 and P45 dendritic shafts, *p < 0.05 for P15 without claws compared to P45). (**f**) Average Gephyrin density on claws (±SEM) in animals of different ages (n = number of claws collected from at least 3 animals per age group).

If claw formation contributes to the stabilization, but not the selection of surviving dendrites, transient synapses on dendritic shafts may play a role in the selection of surviving dendrites. Although claws are the site for both excitatory and inhibitory synapses in mature GCs, previous studies show that GCs receive synaptic inputs from mossy fibers (MFs) even before claws are formed. Postsynaptic densities (PSDs) of immature GCs appear on dendritic shafts, indicating that nascent synapses appear on dendritic shafts before claw formation^17,22^. We, therefore, sought to examine how transient synapses on the shafts affect dendritic refinement. As the first step, we performed immunohistochemical analysis of PSD-95 and Gephyrin-positive puncta on GC dendrites to determine the spatiotemporal pattern of glutamatergic and GABAergic synapse formation (Fig. 5). We confirmed that the puncta of the post-synaptic markers (PSD-95 and Gephyrin) co-localized with GC dendrites and were juxtaposed to pre-synaptic markers (VGLUT for PSD-95 and VGAT for Gephyrin) (Supplementary Fig. S4).

In P45 mice, PSD-95 and Gephyrin puncta were mainly localized on the claws in high density, whereas very few dendritic shafts (4/22 dendrites for PSD-95 and 4/24 for Gephyrin) retain PSD-95 or Gephyrin puncta in low density (P45 in Fig. 5, Supplementary Figs S5a,b,d and S6a,b,d). Therefore, although weak/labile synapses may be present on a few dendritic shafts, synapses are mainly localized at the claws in mature GCs. This is consistent with previous studies, assuring the reliability of our immunohistochemical analysis. The same spatial profile of PSD-95 and Gephyrin puncta was also observed in GCs from P21 mice, suggesting that synapse development on GC dendrites completes by the end of the third postnatal week.

In stark contrast, PSD-95 and Gephyrin puncta were found on both the dendritic shafts, as well as the claws, at high density in younger mice (P10 and P15, Fig. 5). The PSD-95 density on P10 and P15 GC dendritic shafts was four times higher than that on P21 and P45 dendritic shafts (mean PSD-95 puncta density ± SEM: P10/15 = 0.12 ± 0.02, P21/45 = 0.03 ± 0.01, p = 0.002). Similarly, Gephyrin density was also four times higher in P10 and P15 GC dendritic shafts when compared to P21 and P45 dendritic shafts (mean Gephyrin puncta density ± SEM: P10/15 = 0.08 ± 0.01, P21/45 = 0.02 ± 0.01, p < 0.0001). Importantly, not all dendrites in younger mice show similar spatial profiles of PSD-95 and Gephyrin puncta. Dendritic arbors of P10 and P15 GCs consist of immature dendrites without claws and mature dendrites with claws. Only immature dendrites—which tended to have longer shafts than mature dendrites—had significantly more PSD-95 and Gephyrin puncta on shafts than P21 and P45 dendritic shafts (Fig 5b,e, Supplementary Figs S5c, S6c). The mature dendritic shafts (i.e., with claws) of P10 and P15 GCs did not have significantly higher PSD-95 density on shafts when compared to P21 or P45 dendritic shafts, although there was a clear trend (p = 0.05). Furthermore, Gephyrin density on mature dendritic shafts in younger animals (P10, 15) remained significantly higher than adult dendritic shafts (P21, P45) (mean Gephyrin puncta density ± SEM: P10/15 = 0.05 ± 0.01, P21/45 = 0.02 ± 0.01, p = 0.017). With regards to PSD-95 and Gephyrin density on claws, there was no significant difference between age groups (Fig. 5c,f). Additionally, claw size was not significantly different amongst age groups suggesting that once a claw is formed, it does not undergo major growth (Supplementary Figs S5e, S6e). These data suggest that synapses are transiently formed on dendritic shafts of GCs before claws are formed. Maturation of these dendrites must entail a reduction of dendritic shaft length, appearance of claws that marginally increase in size with age, and spatial redistribution of synapses from dendrites to the claw.

### Role of transient synapses in dendritic selection

Unlike mature dendrites with claws, *in vivo* identification of transient synaptic sites is difficult for immature dendrites without claws. Therefore, we sought to identify morphological traits of immature dendrites, such as length and location, which affect their chance of survival and the final pattern of dendritic arbors. This allowed us to determine the extent to which such traits correlate with the number and density of PSD-95 and Gephyrin puncta.

**Figure 6:**
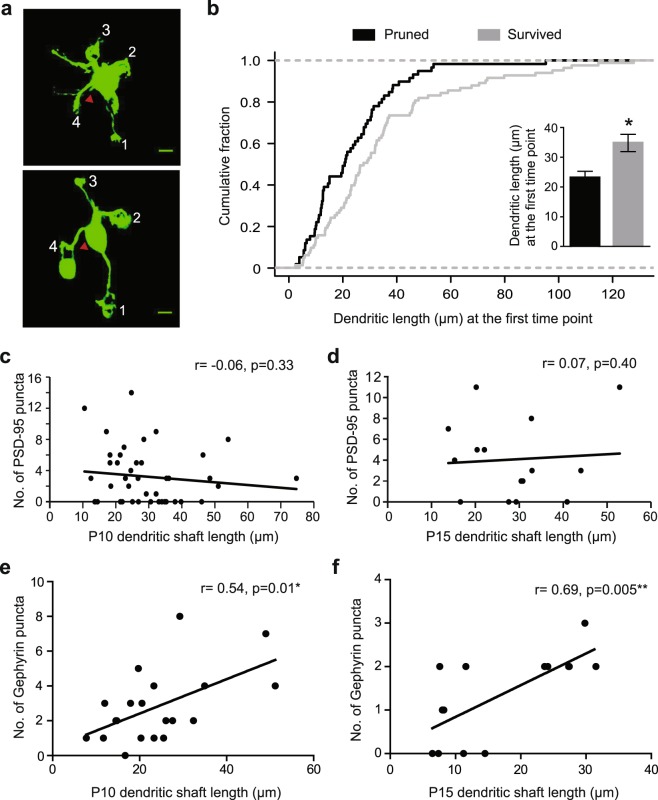
Effect of dendritic length and immature synapse formation on the survival of individual GC dendrites. (**a**) Maximum projection images of a GC at P12 (top) and P21 (bottom). The red arrowhead marks the spot of axon origin. Scale bar is 5 µm. (**b**) Cumulative frequency histogram of dendrites that either survived or were pruned versus their length at the first imaging time point (Kolmogorov–Smirnov test, p = 0.038). The inset shows average dendritic length (±SEM) at the first time point of surviving or pruned dendrites (Unpaired t-test, p = 0.0035). (**c**) Correlation between number of PSD-95 puncta and dendritic shaft length of immature P10 dendrites. (**d**) Correlation between number of PSD-95 puncta and dendritic shaft length of immature P15 dendrites. (**e**) Correlation between number of Gephyrin puncta and dendritic shaft length of immature P10 dendrites. (**f**) Correlation between number of Gephyrin puncta and dendritic shaft length of immature P15 dendrites. N is number of immature dendritic shafts: For PSD-95 correlations, N is 20 for P10 and 21 for P15, For Gephyrin correlations, N is 20 for P10 and 14 for P15. Measurements for (**c**–**f)** were obtained from immunohistochemistry experiments.

Since our data show that the length of immature dendrites affects the dendritic branching pattern of mature GCs (Fig. 3c,d), we examined the relationship between the length of immature dendrites (i.e., without claw) and their eventual fate (i.e., survived or pruned). Surprisingly, the dendrites that survived the refinement process were significantly longer than the dendrites that were eventually pruned when they were at an immature stage (Fig. 6b inset). The average length of surviving dendrites at the first time point was 34.81 ± 2.92 µm, which was about 50% longer than that of pruned dendrites (23.14 ± 2.15 µm). The cumulative frequency histogram of dendritic length at the first time point showed a significant rightward shift for surviving dendrites when compared to the pruned dendrites (Fig. 6b, Kolmogorov–Smirnov test, p = 0.038). These data suggest that the length of immature dendrites affects their eventual fate; longer dendrites are less likely to be pruned than shorter ones.

Since synaptic activity has been shown to be important for dendritic refinement, we hypothesized that longer dendrites have a higher chance of survival because they have more transient synapses on dendritic shafts. To test this hypothesis, we used the immunohistochemistry data (Fig. 5) and determined the correlation between the length of immature dendrites (i.e., without claws) and the number of PSD-95 and Gephyrin puncta on their shafts at P10 and P15. We chose these ages because the first time point of our *in vivo* imaging was between these ages, mostly at P11 and P12. We found that in GCs at both P10 and P15, the length of immature dendrites did not correlate with the number of PSD-95 puncta but was positively correlated with the number of Gephyrin puncta (Fig. 6c–f). Therefore, longer immature dendrites, which have a higher chance of survival, receive more GABAergic inputs on their shafts. Although this is just a correlation, having more GABAergic inputs during the dendritic refinement process might be favorable for the dendrites to survive.

## Discussion

The cerebellar granule cells (GCs) have been studied extensively to understand neuronal migration, dendritic development, and synapse formation^23–26^. In this study, we focused on dendritic refinement, a process in which early-formed redundant dendrites are pruned, whereas necessary dendrites mature to form stable synapses with appropriate presynaptic terminals^7,27^. Our first objective was to determine the exact temporal pattern of GC dendritic refinement *in vivo*. Using two-photon time-lapse microscopy, we analyzed the development of fluorescently-labeled GCs in the TCGO transgenic mouse line that shows sparse expression of mCitrine in these cells. By daily imaging of the same GCs, we revealed that excess dendrites were pruned from P11 to P17, while surviving dendrites matured and developed synapses from P14 until P20 (Supplementary Fig. S1). These data seem to be consistent with previous findings from fixed tissue samples that suggest the stages of dendritic pruning and maturation occur in succession within the same neuron^6,28^. However, it is clear from our time-lapse imaging data that these seemingly distinct stages of dendritic development are occurring simultaneously. While certain dendrites are being pruned, others are growing and developing claws within the same cell. This is evident when we aligned our time-lapse imaging data with reference to the time individual GCs reached maturity (Fig. 2). These differences between individual dendrites could be because individual dendrites might receive different extrinsic cues or respond differentially to the same cues depending on the different distribution of intrinsic factors among each dendrite.

In addition to revealing the pattern of dendritic development in GCs, we also determined that the process of GC dendritic maturation coincides with its stabilization. A mature GC has a claw-like ending on all its dendrites. Using this criterion, we identified the time point for GC maturation. Past this time point, the dendritic arbor of these cells showed minimum changes in total length and no changes in the number of dendrites, indicating stabilization of the arbor (Fig. 2b). This is further corroborated by the result that the change in length of individual dendrites was significantly reduced after the day of claw formation (Fig. 4c,d, Supplementary Fig. 3c,d). Our data obtained from GCs in fixed tissues also support this result, since the length of mature dendrites and claws does not significantly differ between different age groups (Supplementary Figs S5c,e and S6c,e). These data, together with the previous finding that MFs show little to no structural plasticity in adulthood^13^, provide new insight into the stability of GC-MF synapses: no structural rewiring occurs in GC-MF synapses once GCs acquire mature morphology.

The second objective of this study was to understand how specific dendrites are selected for survival and stabilized within the GC arbor. We have found that longer immature dendrites have a higher chance of survival than shorter dendrites, and that immature GCs with longer dendrites tend to be branched GCs. These results suggest that the length of immature dendrites is one of the factors that determine the fate of individual dendrites, as well as the final branching pattern of dendritic arbors. Alternatively, there might not be any mechanism for dendritic selection; i.e., all immature dendrites might naturally shrink during maturation, and shorter dendrites are pruned simply because they are short. However, the latter scenario is highly unlikely. First, the pruning of GC dendrites is not a consequence of gradual shrinkage of immature dendrites. The average length of pruned dendrites was relatively constant with some dendrites even elongating until the day they suddenly disappeared, suggesting that they were pruned by some active mechanisms (Supplementary Fig. 3a,b). Second, MFs are divided into several subclasses based on their synaptic strength with GCs, and more than two strong MFs rarely converge onto the same GC^29^. Such a stereotypic connectivity is hard to emerge if dendritic pruning of GCs is a random process.

The synaptotropic model of neuronal circuit development predicts that synaptic activity is the key cell-extrinsic factor that selects and stabilizes individual developing dendrites. In mouse somatosensory cortex, N-methyl-D-aspartate (NMDA) receptors play a crucial role in the dendritic refinement of layer 4 stellate neurons, such that dendrites oriented toward presynaptic axons are stabilized, whereas others are pruned^30^. However, several recent reports have shown that the loss of presynaptic glutamate release or postsynaptic glutamate receptors did not affect excitatory synapse formation or dendritic arborization in mouse hippocampal pyramidal neurons^31,32^. These studies suggest that the role of synaptic activity in dendritic refinement differs among different neuronal subtypes.

In mature GCs, the claw-like ending of dendrites has been identified as the main site of excitatory and inhibitory synapses. This is consistent with our immunohistochemical analysis of PSD-95 and Gephyrin puncta on GC dendrites that shows a significantly higher density of PSD-95 and Gephyrin puncta on claws than on shafts (Fig. 5). Furthermore, our time-lapse analysis of individual dendrites revealed that claw formation is almost perfectly related to dendritic survival. Out of 84 dendrites that developed a claw, 83 dendrites survived, showing a nearly 100% of survival rate (Fig. 4b). These results appear to support the idea that synapse formation at claws, and maybe synaptic activity too, play an essential role in the selection of surviving GC dendrites and their long-term stabilization. However, analysis of the temporal pattern of dendritic selection suggests that surviving dendrites are selected before claw formation and that claws mainly function to stabilize the preselected dendrites (Fig. 3). Thus, if synaptic activity regulates the selection of surviving dendrites, it must occur in immature dendrites without a claw. This is possible because, as previous studies show, MF-GC and Golgi cell-GC synapses are functional before claw formation peaks in the GC layer^22,33^. In the present study, we have examined the distribution of transient synapses in immature dendrites and found that the number of Gephyrin puncta, but not PSD-95 puncta, positively correlates with dendritic length, which is a morphological factor related to dendritic survival (Fig. 6). At present, it is technically difficult to establish the causal role of GABAergic or glutamatergic inputs in the survival of individual dendrites. Nevertheless, the correlation between dendritic survival and the number of GABAergic inputs prompts us to speculate that there is a potential role of transient GABAergic inputs in dendritic selection. GCs start expressing GABAα receptors as early as P5, and Golgi cells that provide inhibitory inputs to GCs start expressing GABA at P3^34–36^. Similar to many other cell types, GABA may act as an excitatory neurotransmitter in GCs during development because GCs express K1-coupled Cl2 transporter 2 (KCC2), which has been suggested to be responsible for the role of GABA in early neural circuit formation^37,38^. Previous studies in different neuronal types have also observed the role of GABA and GABAergic input on dendritic development and refinement^39,40^.

In conclusion, this study has revealed the temporal pattern of GC dendritic refinement for the first time *in vivo* and starts to elaborate the role of transient synapses found in immature neurons in this process. Initial dendritic selection is influenced by the length of immature dendrites, which might be through transient GABAergic inputs on them. The selected dendrites are subsequently stabilized by the formation of claws, the site of mature synapses on the maturing dendrites.

## Materials and Methods

### Animals

All procedures were approved by the Institutional Animal Care and Use Committee of the University of Texas at Austin. All experiments for this study were conducted in accordance with the institutional guidelines and regulations for animal experiments. Male and female TCGO transgenic mice that express mCitrine fluorescent protein in a small, random subset of cerebellar granule cells were used for this study^14,15^.

### Cranial window surgery

Neonatal mice (6–7 day old, both sexes) were anesthetized with a subcutaneous injection of ketamine/xylazine (50/5 mg/kg), and cranial window surgery was performed as described previously^41^. Briefly, the scalp, muscles, and fascia overlying the skull were removed. A small magnet, glued to the skull near lambda with surgical cyanoacrylate (Vetabond; 3 M) and dental cement, was used to stabilize the animals’ head with clamps attached to the surgical stage. A craniotomy was performed using a 26-gauge needle to etch a small rectangle (2 × 1.5 mm^2^) on the skull over the cerebellar vermis of lobule 6/7. We selected these lobules because previous reports show that GC migration and cortical development occurs last in lobule 6/7^42^. A coverslip was placed directly on top of the dura and secured in place using surgical cyanoacrylate and dental cement. The animal was then allowed to recover from the anesthesia and returned to the home cage.

### *In vivo* imaging

Long-term two-photon *in vivo* time-lapse microscopy was performed as described in our previous publications^43^. Briefly, 4 days (around P10–11) following cranial window surgery, mice were lightly anesthetized with 1–1.5% isoflurane and securely placed on a custom-made microscope stage. The stage was then fixed on a x-y translator under a two-photon laser-scanning microscope (FV1000MPE, Olympus, Tokyo, Japan) equipped with a 25× water immersion objective lens (Olympus XLPlan N, 1.05 NA) and two external gallium arsenide photodetectors (GaAsPs, Hamamatsu, Japan). For two-photon excitation of mCitrine, 900 nm of pulsed infrared laser was provided by Mai Tai HP DeepSee mode-locked Ti:sapphire laser (Spectra-Physics, Santa Clara, CA). The emitted green fluorescent signals were detected by the GaAsPs. The z-stack images (spaced 1 μm apart) of GCs in the cerebellar internal granule cell layer were taken with a resolution of 0.16 µm/pixel in most images. Brain surface vasculature was also imaged in wide-field fluorescence mode to enable us to locate the same GCs in subsequent imaging sessions. The unique pattern of the major brain vessels and sparsity of GCs (fewer than 5 labeled GCs at the earliest imaging sessions) ensured that the same GCs were imaged across every session. After image acquisition, the animals were allowed to recover from the anesthesia and returned to the home cage.

### *In vivo* image analysis and statistics

Images were analyzed using the simple neurite tracer plugin in Fiji, an ImageJ-based open source image processing package (http://fiji.sc/Fiji). GCs were selected at the first time point based on their immature morphology, and the dendrites and claws were traced completely at each time point. Tracing was done on a z-stack of images, and the plugin also kept track of measurements for each dendrite. Twenty-one cells from six TCGO transgenic pups were imaged for the experiments. These sample sizes were not pre-determined by any statistical methods, but were chosen on the basis of what is normally reported in similar long-term *in vivo* time-lapse imaging publications^32,43–45^. No randomization or blinding was necessary since no experimental treatment was applied. For all ANOVAs, normality of data was confirmed using the Shapiro-Wilk normality test, and equality of variance between groups was confirmed using Bartlett’s test. An estimate of variance within each group was calculated and is reported in the relevant figures as error bars (standard error of mean; SEM) and in the results section. All time course data are presented as average ± SEM.

### Immunohistochemistry

For immunohistochemical staining of PSD-95, the method described by Eroglu lab in their previous publications was used^46^. The same method was applied for Gephyrin staining as well. Briefly, animals were anaesthetized with an intraperitoneal injection of ketamine/xylazine (50/5 mg/kg for neonates and 100/10 mg/kg for adults) followed by transcardial perfusion with 4% paraformaldehyde in 0.1 M tris-phosphate buffer saline (TBS: 25 mM Tris-base, 135 mM NaCl, 3 mM KCl, pH 7.6). Post-perfusion, the brain was extracted and further fixed overnight by immersion fixation in 4% paraformaldehyde in 0.1 M TBS at 4 °C. Next, brains were submerged in 30% sucrose for cryoprotection overnight at 4 °C and then embedded in OCT (Fischer-Brand) before cryosectioning using Leica CM3050 cryostat at 20 µm thickness. Sections were washed and permeabilized in TBS with 0.2% Triton X-100 (TBST) and then blocked in 5% normal goat serum (NGS) in TBST for 1 h at room temperature. Sections were incubated overnight at 4 °C with anti-PSD-95 antibodies (1:350 diluted in 5% NGS in TBST, Invitrogen 51–6900) or anti-Gephyrin antibodies (1:1500 diluted in 5% NGS in TBST, Synaptic systems 147–011) followed by secondary antibody (1:500 Cy5 diluted in 5% NGS in TBST, Jackson 111-175-144) incubation for 1 hour at room temperature. After washing, slices were mounted (Permafluor, Thermo Fisher Scientific, Waltham, MA), coverslipped and imaged using a laser-scanning confocal microscope (FV-1000, Olympus, Tokyo, Japan). Z-stack of images (0.1 µm^2^) were acquired at 0.33 µm step-size using 515 nm and 635 nm lasers. Three TCGO animals were used per age group and GCs from at least two sagittal brain sections were imaged per animal. For P10 animals, 64 dendrites and 19 claws from 21 GCs; for P15 animals, 36 dendrites and 21 claws from 14 GCs; for P21 animals, 24 dendrites and claws from 12 GCs; and for P45 animals 22 dendrites and claws from 19 GCs were imaged and analyzed for PSD-95 analysis. For P10 animals, 35 dendrites and 18 claws from 13 GCs; for P15 animals, 34 dendrites and 20 claws from 10 GCs; for P21 animals, 23 dendrites and claws from 14 GCs; and for P45 animals 24 dendrites and claws from 8 GCs were imaged and analyzed for Gephyrin analysis. For pre-synaptic markers, anti-VGLUT (1:1000 diluted in 5% NGS in TBST, Synaptic systems 135–421) and anti-VGAT (1:1000 diluted in 5% NGS in TBST, Synaptic systems 131–003) primary antibodies were used along with the post-synaptic antibodies to stain sections obtained from P45 animals. The secondary used for pre-synaptic markers was 1:500 Alexa 405.

### Puncta analysis and statistics

For measurements of synaptic puncta on dendrites and claws, the following Fiji plugins were used: ROI manager, analyze particles, simple neurite tracer. Z-stack of images containing both channels (channel 1 = 515 nm for GC, channel 2 = 635 nm for PSD-95/Gephyrin) were imported into Fiji. After splitting channels, channel 1 was used to define ROIs (in 3D) around dendrites that were clearly emerging from a soma and around claws attached to those dendrites. Care was taken to define the ROI as closely as possible around these structures (see Fig. 5a,d) since the internal granule cell layer is densely packed with GCs and their synapses, especially in adult animals. All the ROIs from channel 1 were saved in the ROI manager, to be used to measure the number of puncta within those ROIs in channel 2. Before puncta measurement, the z-stack of channel 2 images were modified to create a binary image using threshold adjustment (Fiji preset “IsoData” applied to all images uniformly). The modified z-stack image was then inverted such that PSD-95/Gephyrin staining appeared black and the background was white. This inverted image was then used to measure the number of puncta, using the analyze particles plugin, within the ROIs traced in corresponding channel 1 images which measures puncta in each plane of a z-stack separately. In the analyze particles plugin, circularity was set to 0 (i.e., no circularity was expected) and minimum puncta size was set to 0.5 µm. All the puncta identified by the analyzer were added to the ROI manager, and this was used to manually confirm that each puncta actually overlaps with the dendritic/claw signal in channel 1. The length of dendrites and claws used to generate ROIs were measured using the simple neurite tracer which can efficiently measure neurite lengths in 3D images. Statistical tests used to compare different age groups are mentioned in appropriate figure legends and data are displayed as mean ± SEM.

## Electronic supplementary material

Supplementary Information

## Data Availability

The datasets generated and analyzed during the current study are available from the corresponding author on reasonable request.
