# Supplementary material for: Developmental pattern and structural factors of dendritic survival in cerebellar granule cells in vivo
Source: Sci Rep. 2018 Dec 3;8:17561. doi: 10.1038/s41598-018-35829-y (PMC6277421; doi:10.1038/s41598-018-35829-y)
Supplement: Supplementary file 1 — Supplementary Information [file 41598_2018_35829_MOESM1_ESM.pdf]

Supplementary Information

Developmental pattern and structural factors of dendritic survival in cerebellar granule cells *in vivo*

Matasha Dhar[1], Adam W. Hantman[2], Hiroshi Nishiyama[1,] *
[1]Center for Learning and Memory, Department of Neuroscience, The University of Texas at Austin, 1 University Station Stop C7000, Austin, Texas
[2]Janelia Research Campus, Howard Hughes Medical Institute, Ashburn, Virginia

*Corresponding author
E-mail: hiroshi@utexas.edu
Telephone: 512-232-8438
Fax: 512-475-8000

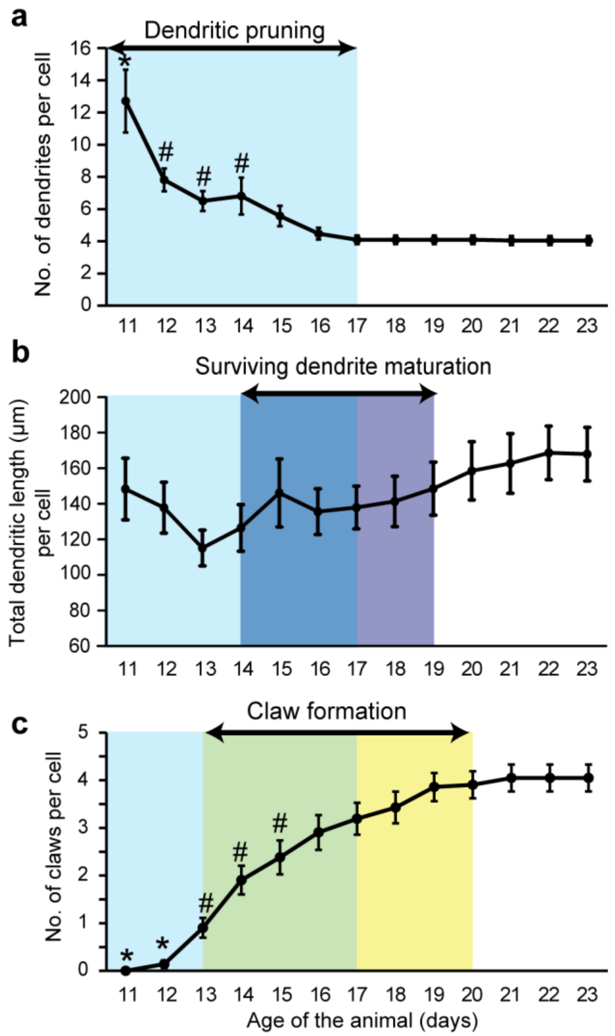

Supplementary figure S1 (with Figure 1): Pattern of GC dendritic development *in vivo* with respect to animal age. (a) Average number of total dendrites per cell (±SEM) at each time point (n=21 cells from 6 mice). The blue box highlights the dendritic pruning phase for the entire population of GCs imaged. Number of dendrites per cell were significantly different over time (One-way ANOVA with Tukey post-hoc analysis: $F(12, 238) = 11.24$, $p<0.0001$, *$p<0.001$ for time point 11 compared with all other time points, #$p<0.01$ for time points 12, 13, 14 compared with time points 17-23). **(b)** Average total dendritic length per cell (±SEM) at each time point. The blue box highlights the dendritic pruning phase (from panel **a**) and the purple box highlights the dendritic maturation phase for the entire population of GCs imaged. **(c)** Average number of claws per cell (±SEM) at each time point. The blue box highlights the dendritic pruning phase (from panel **a**) and the yellow box highlights the claw formation phase for the entire population of GCs imaged. Number of claws per cell were significantly different over time (One-way ANOVA with Tukey analysis: $F(12, 260) = 28.35$, $p< 0.0001$, *$p<0.001$ for time points 11, 12 compared with time points 14-23, #$p<0.01$ for time point 13 compared with time points 16-23, #$p<0.05$ for time points 14, 15 compared with time points 19-23).

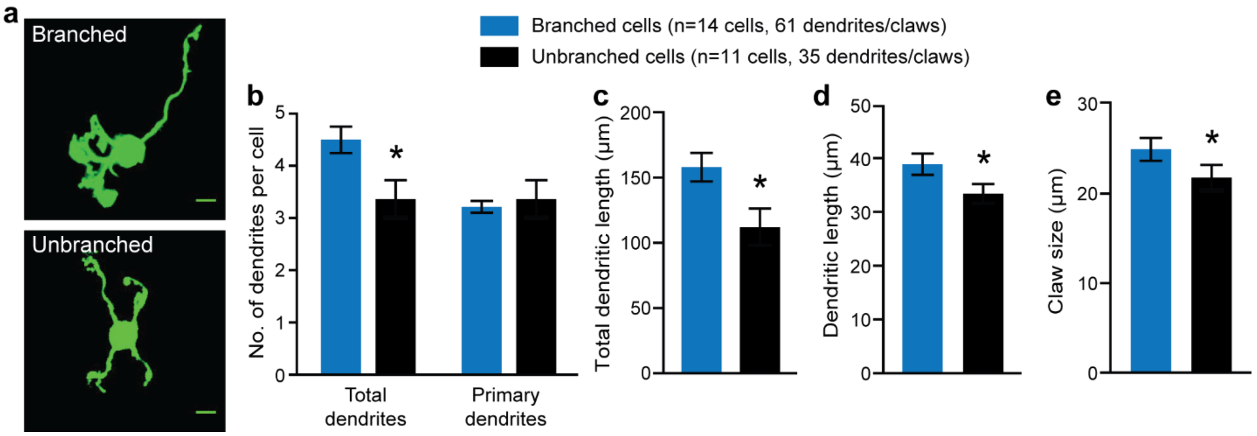

Supplementary figure S2 (with Figure 3): Differences between mature branched and unbranched GCs. (a) Maximum projection image of P23 GCs with branched or only unbranched dendrites. Scale bar is 5µm. **(b)** Average number of total or primary dendrites (±SEM) for cells with at least one set of branched dendrites (branched cells, blue) and cells with only unbranched dendrites (unbranched cells, black). Number of total dendrites per cell is significantly different between the two categories of cells (Unpaired t-test, $p=0.014$). **(c)** Average total dendritic length (±SEM) for branched cells and unbranched cells. The total length of dendrites is significantly different between the two categories of cells (Unpaired t-test, $p=0.015$). **(d)** Average length of individual dendrites (±SEM) for branched cells and unbranched cells. Length of individual dendrites is significantly different between the two categories of cells (Unpaired t-test, $p=0.031$). **(e)** Average length of individual claws (±SEM) for branched cells and unbranched cells. Length of claws is significantly different between the two categories of cells (Unpaired t-test, $p=0.045$).

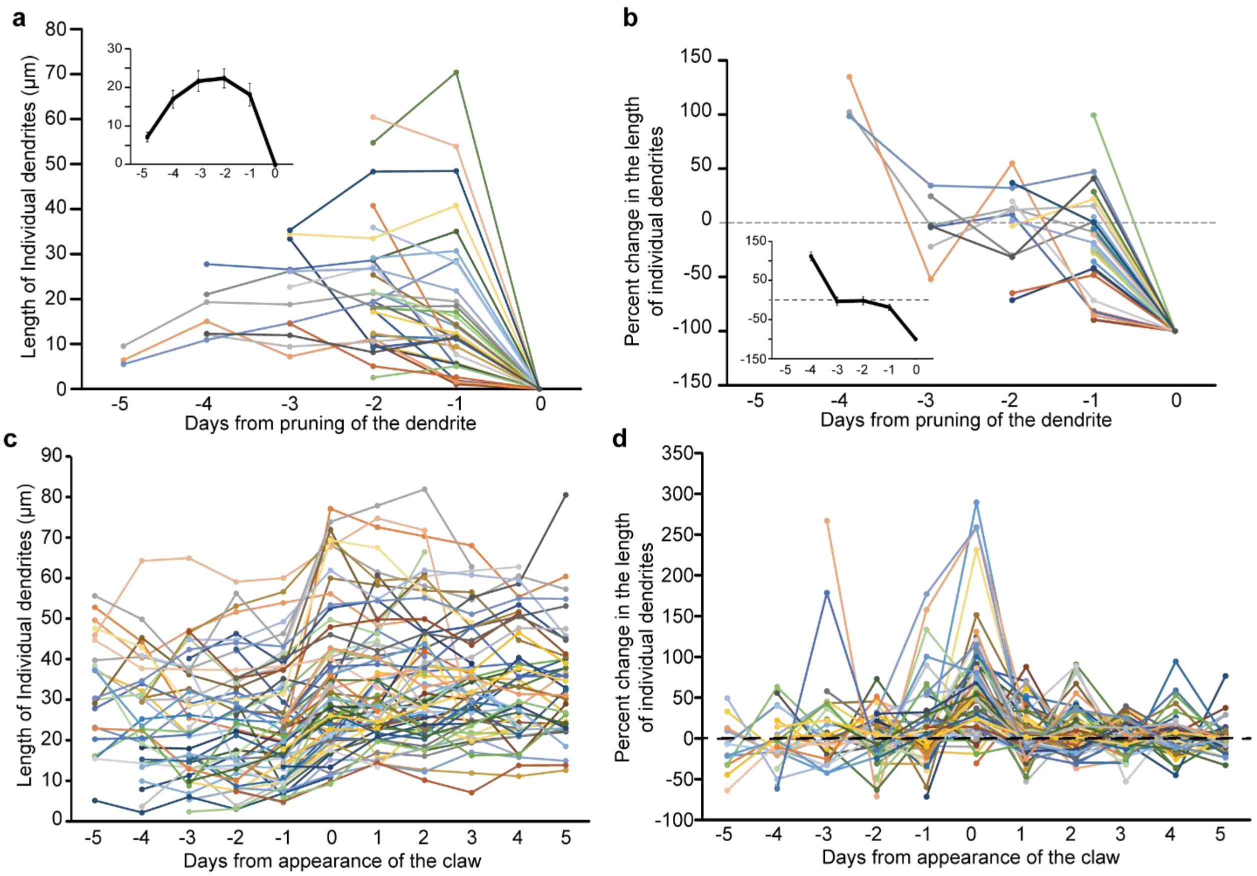

Supplementary figure S3 (with Figure 4): Dynamic, time-lapse changes of individual dendrites (a) Length of pruned dendrites at each time point with time point 0 being the day the dendrite was pruned. Inset: Average length of individual pruned dendrites (±SEM) at each time point with time point 0 being the day the dendrite was pruned**. (b)** Percent change in the length of pruned dendrites at each time point with time point 0 being the day the dendrite was pruned. Inset: Average percent change in the length of individual pruned dendrites (±SEM) at each time point with time point 0 being the day the dendrite was pruned**. (c)** Length of individual dendrites measured for Figure 4c at each time point with time point 0 being the day a claw was observed on a dendrite. Average length is shown in Figure 4c, red line. **(d)** Percent change in the length of individual dendrites measured for figure 4c at each time point with time point 0 being the day a claw was observed on a dendrite. Average percent change is shown in Figure 4c, blue line.

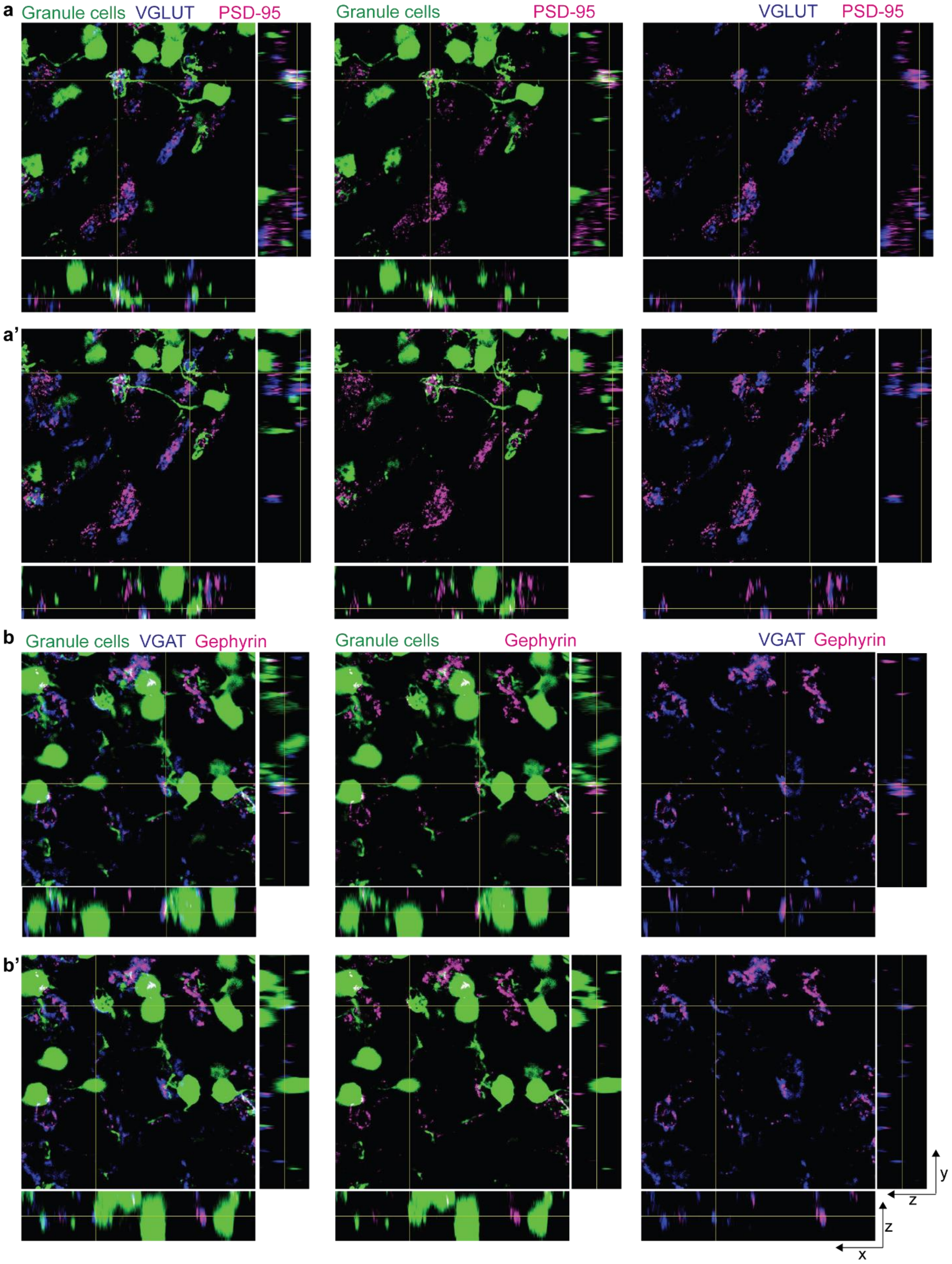
a
Granule cells VGLUT PSD-95
Granule cells PSD-95
VGLUT PSD-95
a'
b
Granule cells VGAT Gephyrin
Granule cells Gephyrin
VGAT Gephyrin
b'
y
z
z
x

Supplementary figure S4 (with Figure 5): Immunohistochemical analysis of excitatory and inhibitory synapses on GC dendrites. (a, a’, b, b’) Representative images of GCs from adult animals showing GCs, postsynaptic marker (a, a’: PSD-95; b, b’: Gephyrin) and a presynaptic marker (a, a’: VGLUT; b, b’: VGAT) simultaneously in the first column. The second column shows the co-localization between GC dendrites and postsynaptic markers (a, a’: PSD-95; b, b’: Gephyrin). The third column shows the juxtaposition between presynaptic markers (a, a’: VGLUT; b, b’: VGAT) and postsynaptic markers. In each group of images, the central image is a single x-y plane with the crosshairs marking the same spot that is then shown in both x-z and y-z profiles. Images in panels a and a’ show two separate claws clearly visible and located in the same x-y plane in the same central image and their juxtaposition with the marker used. Same for panel b and b’.

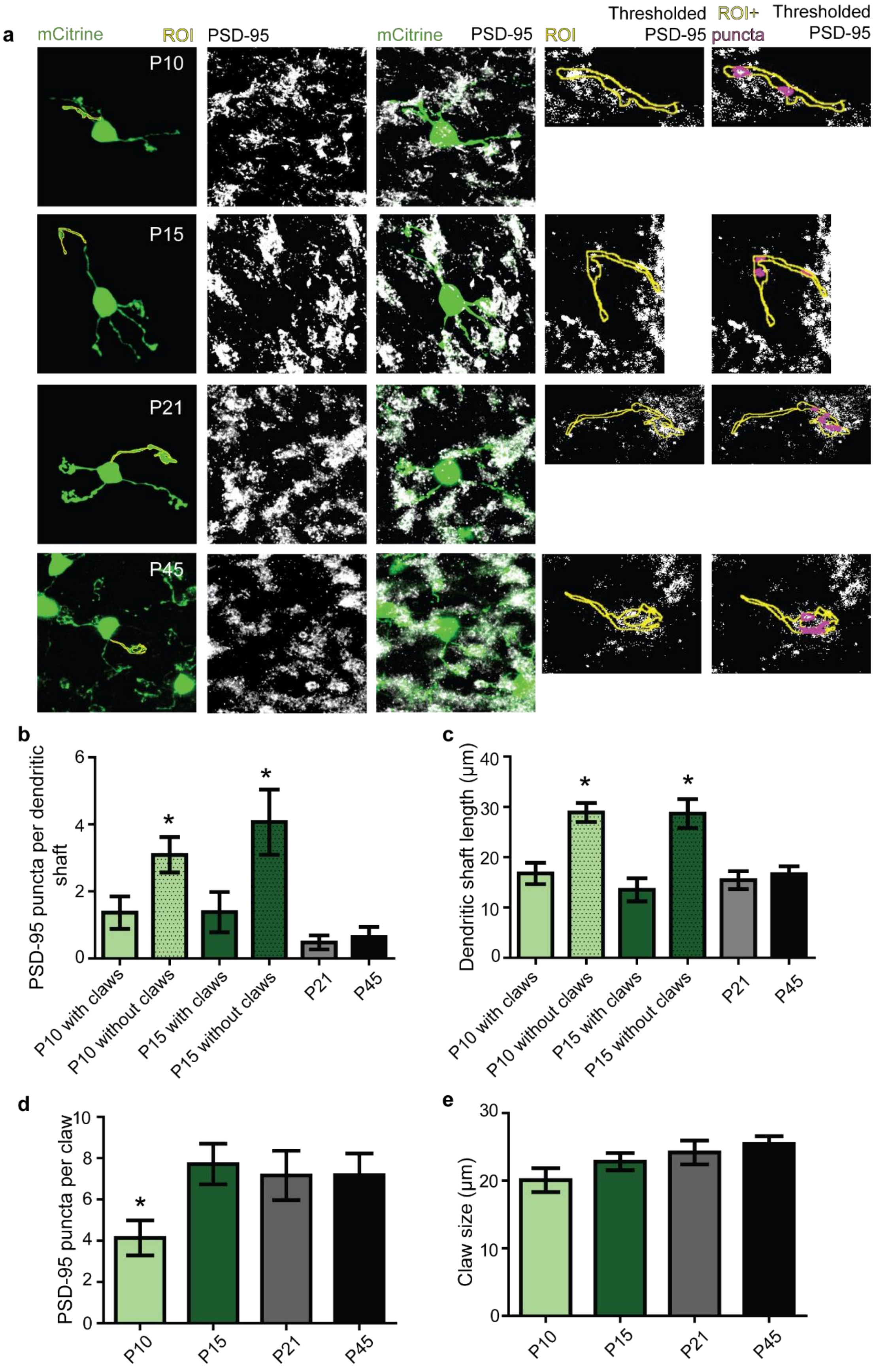

a
mCitrine
ROI
PSD-95
mCitrine
PSD-95
ROI
Thresholded PSD-95
ROI+ puncta
Thresholded PSD-95
P10
P15
P21
P45
b
PSD-95 puncta per dendritic shaft
P10 with claws
P10 without claws
P15 with claws
P15 without claws
P21
P45
c
Dendritic shaft length (µm)
P10 with claws
P10 without claws
P15 with claws
P15 without claws
P21
P45
d
PSD-95 puncta per claw
P10
P15
P21
P45
e
Claw size (µm)
P10
P15
P21
P45

Supplementary figure S5 (with Figure 5): Immunohistochemical analysis of the developmental pattern of excitatory synapse formation on GC dendrites. (a) From the left, maximum projection images of GCs (mCitrine, green), PSD-95 staining (white), overlay of GCs and PSD-95 staining, and single focal plane images showing PSD-95 puncta (magenta outlines) in the outlines of GC dendrites that are within the same focal plane (yellow ROIs). These images are for the cells shown in Figure 5a. Maximum projection images lack information along the z-axis; thus, the colocalization between GC dendrites and PSD-95 puncta cannot be assessed. Therefore, the colocalization of PSD-95 puncta on exemplary dendrites (yellow ROIs in GC images) is shown in the single focal plane by overlaying the ROIs with the thresholded PSD-95 signals. Although each ROI covers a single dendritic segment in this figure, it is to explain our analytical method. Entire dendrites of individual GCs were analyzed. See Materials and Methods for automatic thresholding of PSD-95 signals and detection of the puncta. **(b)** Average number of PSD-95 puncta on dendritic shafts (±SEM) in animals of different ages (n = number of dendritic shafts collected from at least 3 animals per age group). Number of PSD-95 puncta is significantly different amongst dendritic shafts of different age groups (Kruskal-Wallis non-parametric ANOVA, $H(6) = 27.63$, $p < 0.0001$, $^*p<0.01$ for P10 and P15 dendritic shafts without claws compared to P21 and P45 dendritic shafts (all with claws). **(c)** Average length of dendritic shafts (±SEM) in animals of different ages (n = number of dendritic shafts collected from at least 3 animals per age group). Length of dendritic shafts is significantly different amongst different age groups (Kruskal-Wallis non-parametric ANOVA, $H(6) < 0.0001$, $^*p<0.01$ for P10 and P15 dendritic shafts without claws compared to P21 and P45 dendritic shafts (all with claws). **(d)** Average number of PSD-95 puncta on claws (±SEM) in animals of different ages (n = number of dendrites collected from at least 3 animals per age group). Number of PSD-95 puncta is significantly different amongst claws of different age groups (Kruskal-Wallis non-parametric ANOVA, $H(4) = 7.935$, $p=0.048$, $^*p<0.05$ for P10 claws compared to P15, P21, and P45 claws). **(e)** Average length of claws (±SEM) in animals of different ages (n = number of claws collected from at least 3 animals per age group).

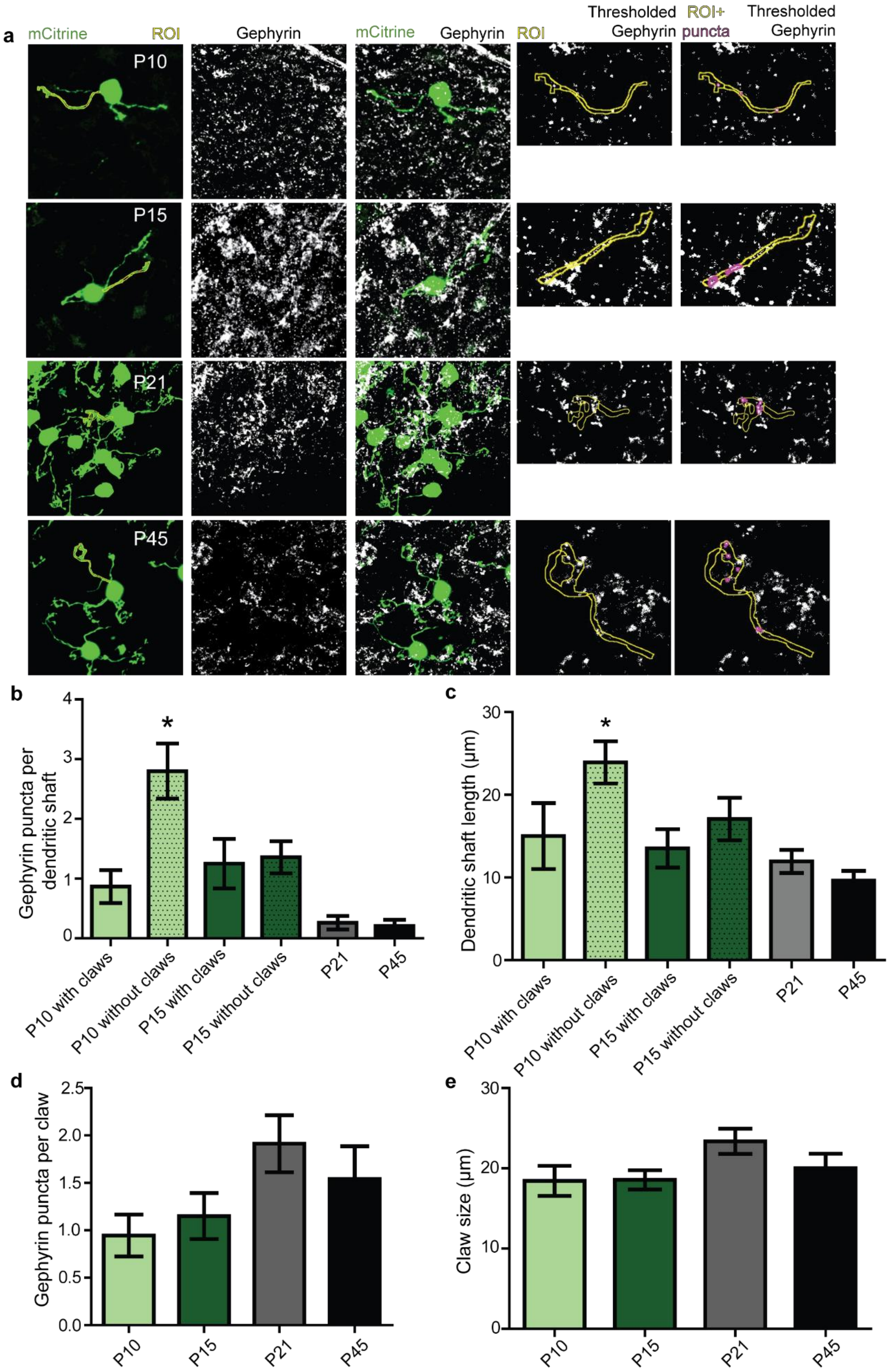

a
mCitrine
ROI
Gephyrin
mCitrine
Gephyrin
ROI
Thresholded Gephyrin
ROI+ puncta
Thresholded Gephyrin
P10
P15
P21
P45
b
Gephyrin puncta per dendritic shaft
P10 with claws
P10 without claws
P15 with claws
P15 without claws
P21
P45
c
Dendritic shaft length (µm)
P10 with claws
P10 without claws
P15 with claws
P15 without claws
P21
P45
d
Gephyrin puncta per claw
P10
P15
P21
P45
e
Claw size (µm)
P10
P15
P21
P45

Supplementary figure S6 (with Figure 5): Immunohistochemical analysis of the developmental pattern of inhibitory synapse formation on GC dendrites. (a) From the left, maximum projection images of GCs (mCitrine, green), Gephyrin staining (white), overlay of GCs and Gephyrin staining, and single focal plane images showing Gephyrin puncta (magenta outlines) in the outlines of GC dendrites that are within the same focal plane (yellow ROIs). These images are for the cells shown in Figure 5d. The colocalization of Gephyrin puncta on exemplary dendrites (yellow ROIs in GC images) is shown in the single focal plane by overlaying the ROIs with the thresholded Gephyrin signals. **(b)** Average number of Gephyrin puncta on dendritic shafts (±SEM) in animals of different ages (n = number of dendritic shafts collected from at least 3 animals per age group). Number of Gephyrin puncta is significantly different amongst dendritic shafts of different age groups (Kruskal-Wallis non-parametric ANOVA, $H(6) = 44.12$, $p < 0.0001$, *$p<0.01$ for P10 dendritic shafts without claws compared to P10 with claws, P15 with claws, P21 and P45 dendritic shafts. **(c)** Average length of dendritic shafts (±SEM) in animals of different ages (n = number of dendritic shafts collected from at least 3 animals per age group). Length of dendritic shafts is significantly different amongst different age groups (Kruskal-Wallis non-parametric ANOVA, $H(6) = 23.34$, $p = 0.0003$, *$p<0.01$ for P10 dendritic shafts without claws compared to P10 with claws, P15 with claws, P21, and P45 dendritic shafts. **(d)** Average number of Gephyrin puncta on claws (±SEM) in animals of different ages (n = number of dendrites collected from at least 3 animals per age group). Number of Gephyrin puncta are NOT significantly different amongst claws of different age groups (Kruskal-Wallis non-parametric ANOVA, $H(4) = 5.626$, $p=0.14$). **(e)** Average length of claws (±SEM) in animals of different ages (n = number of claws collected from at least 3 animals per age group).
